# Short-term consumption of a high-fat diet increases host susceptibility to *Listeria monocytogenes* infection

**DOI:** 10.1186/s40168-019-0621-x

**Published:** 2019-01-18

**Authors:** Vanessa Las Heras, Adam G. Clooney, Feargal J. Ryan, Raul Cabrera-Rubio, Pat G. Casey, Cara M. Hueston, Jorge Pinheiro, Justine K. Rudkin, Silvia Melgar, Paul D. Cotter, Colin Hill, Cormac G. M. Gahan

**Affiliations:** 1 APC Microbiome Ireland, University College Cork, Cork, Ireland; 2 School of Microbiology, University College Cork, Cork, Ireland; 3 Teagasc Food Research Centre, Moorepark, Fermoy, Cork, Ireland; 4 School of Pharmacy, University College Cork, Cork, Ireland

**Keywords:** Diet, Infection, Microbiome, *Listeria monocytogenes*, Mice, Murine, Immunity, Goblet cell

## Abstract

**Background:**

A westernized diet comprising a high caloric intake from animal fats is known to influence the development of pathological inflammatory conditions. However, there has been relatively little focus upon the implications of such diets for the progression of infectious disease. Here, we investigated the influence of a high-fat (HF) diet upon parameters that influence *Listeria monocytogenes* infection in mice.

**Results:**

We determined that short-term administration of a HF diet increases the number of goblet cells, a known binding site for the pathogen, in the gut and also induces profound changes to the microbiota and promotes a pro-inflammatory gene expression profile in the host. Host physiological changes were concordant with significantly increased susceptibility to oral *L. monocytogenes* infection in mice fed a HF diet relative to low fat (LF)- or chow-fed animals. Prior to *Listeria* infection, short-term consumption of HF diet elevated levels of *Firmicutes* including *Coprococcus*, *Butyricicoccus*, *Turicibacter* and Clostridium XIVa species. During active infection with *L. monocytogenes*, microbiota changes were further exaggerated but host inflammatory responses were significantly downregulated relative to *Listeria*-infected LF- or chow-fed groups, suggestive of a profound tempering of the host response influenced by infection in the context of a HF diet. The effects of diet were seen beyond the gut, as a HF diet also increased the sensitivity of mice to systemic infection and altered gene expression profiles in the liver.

**Conclusions:**

We adopted a systems approach to identify the effects of HF diet upon *L. monocytogenes* infection through analysis of host responses and microbiota changes (both pre- and post-infection). Overall, the results indicate that short-term consumption of a westernized diet has the capacity to significantly alter host susceptibility to *L. monocytogenes* infection concomitant with changes to the host physiological landscape. The findings suggest that diet should be a consideration when developing models that reflect human infectious disease.

**Electronic supplementary material:**

The online version of this article (10.1186/s40168-019-0621-x) contains supplementary material, which is available to authorized users.

## Background

Increased consumption of a ‘westernized’ diet, comprising high caloric intake from fats and reduced consumption of fermentable fibre, has been linked to the current pandemic of chronic inflammatory conditions such as obesity, type 2 diabetes, inflammatory bowel disease and allergic asthma [1]. A diet rich in animal-derived fats can reduce gastrointestinal barrier function, influence microbiota composition and alter gastrointestinal and systemic inflammatory responses [1–3]. Such profound physiological responses in the host are likely to underpin pathological changes, particularly at mucosal surfaces [1]. However, the potential for a high-fat, westernized diet to influence the progression of infectious disease has received relatively little attention. We proposed to investigate this phenomenon using *Listeria monocytogenes*, a foodborne pathogen that causes a serious invasive disease (listeriosis) in susceptible hosts, is increasingly associated with large common-source outbreaks of disease and has a high mortality rate.

*L. monocytogenes* has been extensively investigated as a model intracellular pathogen to uncover the biological mechanisms involved in host cell invasion, intracellular parasitism and resultant host immunity [4]. The majority of such studies have utilized cell culture models or systemic murine infection. However, an increasing number of studies have begun to focus upon the gastrointestinal phase of infection. It is clear that the pathogen can sense and respond to both local physico-chemical signals [5–7] and the presence of autochthonous organisms [6] in the gastrointestinal environment, and environmental adaptation is likely to influence the ability of the pathogen to survive and transiently replicate in the intestine [5, 7]. Subsequent invasion of the host is through interaction between microbial internalin A (InlA) and host E-cadherin (E-cad), a process which is most efficient in the vicinity of goblet cells where E-cad is more likely to be accessible [8]. In addition, there is a role for the microbiota in providing a barrier to infection and in modifying the host immune response to the pathogen locally [6, 9–11].

We employed a systems approach to study the effects of a HF diet upon a number of parameters associated with the infectious process both before and after infection with *L. monocytogenes*. In particular, we examined both microbiota and host physiological changes influenced by diet both immediately prior to infection and also during the peak period of active infection. Our findings indicate that a relatively short-term change in diet, to a westernized HF diet, increases susceptibility to oral infection with *L. monocytogenes* concomitant with a significantly altered physiological landscape in both the gastrointestinal tract and the liver. Furthermore, our findings show that diet influences the systemic phase of infection alone suggesting a profound system-wide alteration to host physiology that alters susceptibility to infection.

## Results and discussion

### High-fat diet increases susceptibility to oral *L*. *monocytogenes* infection

**Fig. 1:**
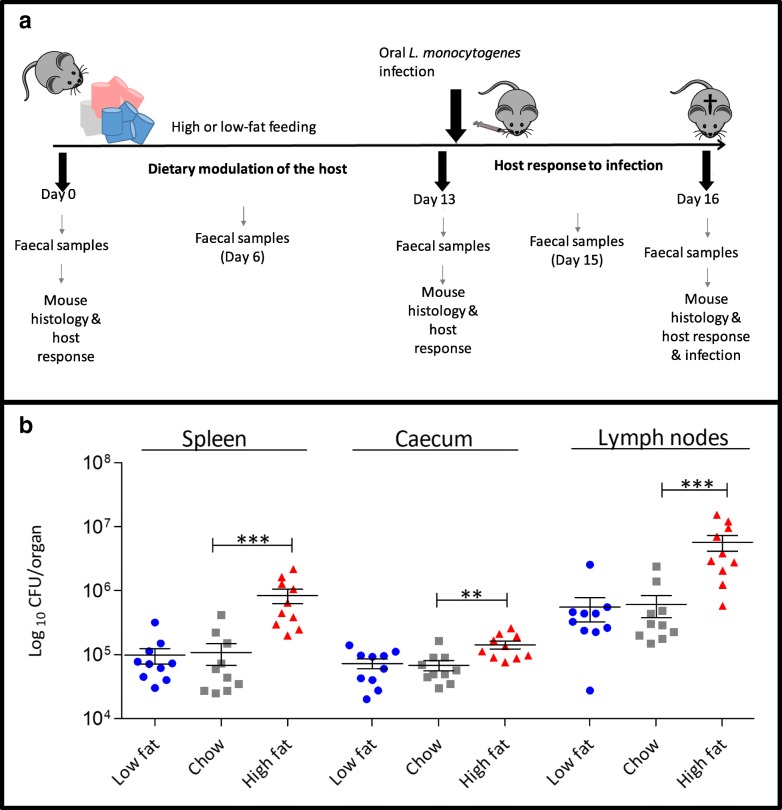
Effect of HF westernized diet on *Listeria monocytogenes* infection in mice. **a** Study overview. C57BL/6 mice (*n* = 10) were fed with a low-fat diet (10% fat), chow (18% fat) and high-fat diet (45% fat) for 13 days, orally infected with 5 × 10^9^ *L. monocytogenes EGDe*^*m*^ and infection determined at 72 h post-infection (day 16). Sampling points for faecal microbiota analysis during dietary modulation of the host and during infection are indicated. Animals were euthanized, and the total number of *L. monocytogenes EGDe*^*m*^ CFU per organ was determined by plating homogenized organs. The phase from D0 to D13 represents the influence of diet upon the host and microbiota whereas D13 to D16 represents a 3-day infection with *L. monocytogenes*. **b** Increased dietary fat increases host susceptibility to oral infection with *L. monocytogenes* EGDe^m^. *Listeria* burden in the spleen, cecum and mesenteric lymph nodes of C57BL/6 mice fed with diets varying in percentage of fat content (*n* = 10, standard deviation from the mean, statistical analysis was conducted using one-way ANOVA and Dunnett’s multiple comparison test in relation to chow diet) **p < 0.01, ***p < 0.001. Error bars represent SEM

We established a study design (Fig. 1a) in which C57Bl/6J mice were fed either a HF diet (45% of the total caloric intake from fat), a matched low-fat (LF) diet (10% of the total caloric intake from fat) or regular chow (18% of the total caloric intake from fat) (Additional file 1: Figure S1a). Feeding was for 2 weeks in order to avoid alterations in systemic fat deposition (obesity) and metabolism associated with longer-term feeding in this model [12]. Indeed, murine body weights were comparable across the different groups after switching diets for 2 weeks (Additional file 1: Figure S1b). At day 13, mice were infected perorally with a strain of *L. monocytogenes* (designated EGDe^m^) in which the InlA protein has been altered to enhance interaction with murine E-cad, thereby increasing the efficacy of the model as a measure of invasive disease [13, 14]. It is known that wild-type *L. monocytogenes* InlA interacts poorly with murine E-cad [4], most likely translocates passively at Peyer’s patches in non-permissive models [15] and is incapable of significant invasive disease in normal mice. We, and others, recognize the limitations of both models [10] and appreciate that the altered InlA expressed in *L. monocytogenes* EGDe^m^ in addition to enhancing interaction with E-cadherin may also interact with murine N-cadherin (mN-cad) [16]. However, the murine model is also reflective of InlA-E-cad-independent pathways used by *L. monocytogenes* to invade and translocate across the intestine [17] and this invasive mechanism is likely to be relevant to human infection [18, 19].

Feeding of HF diet for 2 weeks significantly increased susceptibility to oral *L. monocytogenes* EGDe^m^ infection compared to LF- or chow-fed animals, as indicated by increased levels of the pathogen in internal organs and caecum at day 3 post-infection (Fig. 1b). Faecal levels of the pathogen are indicated in Additional file 2: Figure S2. Repeat experiments utilising an engineered bioluminescent strain of *L. monocytogenes* EGDe^m^ showed similar results (Additional file 2: Figure S2). The data indicate a robust influence of diet upon susceptibility to oral *L. monocytogenes* EGDe^m^ infection in a murine model.

### Dietary modulation of host physiology prior to infection

Diet is known to influence the physiology of the host, and significant research has focused upon the changes that are associated with the onset of obesity in mice fed a HF diet [20]. In contrast, relatively few studies have investigated the gastrointestinal or systemic changes that occur prior to the onset of obesity in this model [21]. We therefore investigated the influence of different diets upon the physiological response of mice following 2 weeks of dietary intervention, immediately prior to oral infection with *L. monocytogenes*. This represents an index of the immediate environment into which *Listeria* is introduced and must establish early infection. We particularly focused upon parameters that are known to play a role in the pathogenesis of *L. monocytogenes*.

**Fig. 2:**
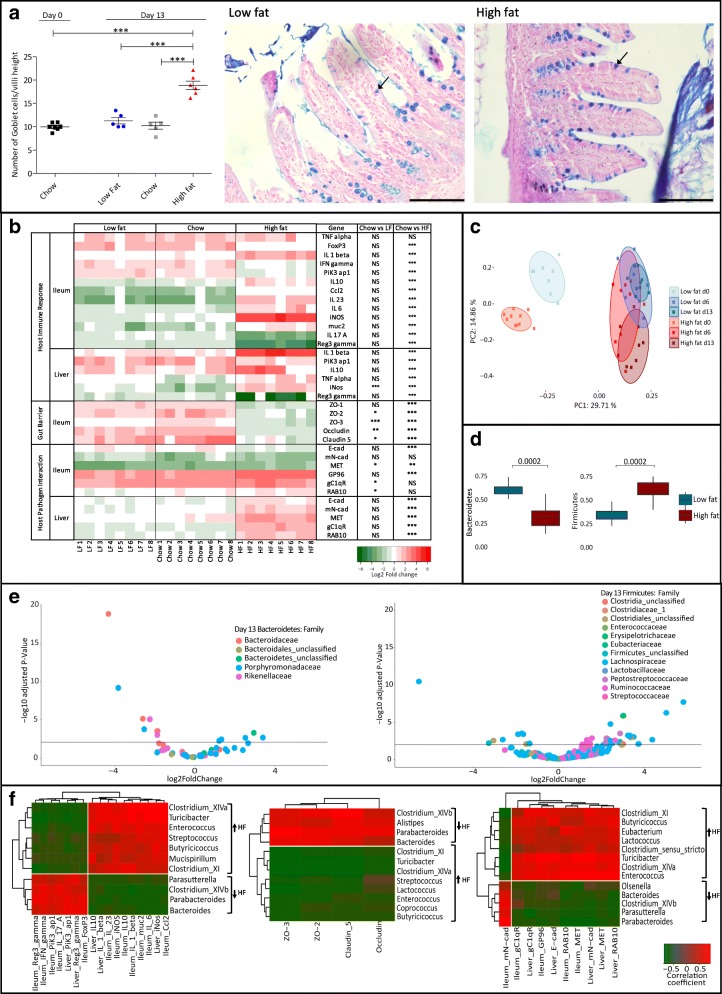
Diet influences the host physiological landscape prior to infection (D0 to D13). **a** Number of goblet cells (D13) present on one villus was quantified and divided by the villus length (groups were blinded). Statistical analysis was conducted using one-way ANOVA and Dunnett’s multiple comparison test in relation to D0. Representative histological images demonstrating goblet cell density (left representative of LF group and right representative of HF group on day D13) (bar 200 μm). Ileal paraffin sections of 5 μm were stained with alcian blue and periodic acid-Schiff (PAS) and counterstained with Schiff reagent and Nuclear Fast Red solution. Arrows indicate examples of goblet cells. **b** Murine gene expression profile in response to increased dietary fat content by qRT-PCR. Represented as log_2_ of the fold change between the condition and the control (D0). Statistical analysis was conducted using one-way ANOVA and Dunnett’s multiple comparison test. **c** Comparison of the changes in bacterial communities during controlled feeding. Unweighted Unifrac PCoA faecal microbiota distances between groups of mice fed different diets (blue representing LF; red representing HF) prior to infection (time points D0, D6, D13). *p* values were measured using an Adonis test (*p* value = 1e−5, *R*^2^ (proportion of variance explained) is 0.48). **d** Changes in bacterial abundance during dietary shifts (D0 to D13) for the most abundant phyla, *Bacteroidetes* and *Firmicutes*. **e** Changes in bacterial abundance between LF and HF diets at the family level for the most abundant phyla. A volcano plot showing the fold change between HF and LF diets at D13. Each point represents an operational taxonomic unit (OTU). The *x*-axis represents the log_2_ of the fold change whilst the *y*-axis is the negative log_10_ of DESeq2 *p* values adjusted for multiple testing using the false discovery rate method. Points to the right of the plot with positive log2FoldChange values represent bacterial taxa with increased abundance in the mice on the HF diet relative to the mice on the LF diet and those with negative log2FoldChange values represent bacterial taxa with increased abundance in the LF diet relative to the HF diet. The horizontal black line represents the cutoff for statistical significance, an adjusted *p* value of 0.05. **f** A correlation between the host regulatory response and the microbiota at the genus level on D13. Spearman correlation, between the diet-dependent relative abundance of bacterial genera (arrows represent abundance in the mice on the HF diet relative to the mice on the LF diet) and the fold change for genes in both ileum and liver. Results shown separately for genes associated with host immunity, tight junction proteins and host-pathogen interaction. Represented are only significant hits, *p* < 0.05. Error bars represent SEM

Blinded histological analysis indicated a significant increase in intestinal goblet cell numbers in mice fed a HF diet relative to both LF- and chow-fed groups (Fig. 2a). Our data suggest an early response of the gut to HF diet feeding that involves generation of goblet cells, and support previous studies showing elevated goblet cell numbers associated with the onset of obesity albeit at a much later stage of HF dietary feeding [22]. As goblet cells are a preferential site of invasion by *L. monocytogenes*, including *L. monocytogenes* strains expressing murinized InlA (the model used in this study) [8, 16], the elevated goblet cell numbers seen in our system are likely to, at least in part, enable enhanced infection.

Transcriptional analysis of a range of relevant host markers was used to determine regulatory changes in the host that occurred between day 0 (the initiation of dietary intervention) and day 13 (just prior to *L. monocytogenes* infection). The host transcriptional profile was similar when comparing animals fed a commonly used mouse chow and animals fed the LF diet. However, HF dietary feeding significantly altered the pattern of gene transcription in mice (Fig. 2b). Generally, mice fed a HF diet demonstrated elevated inflammatory gene expression relative to the LF- and chow-fed mice. The data are consistent with the concept that a HF diet promotes a pro-inflammatory state in the host and are supportive of longer-term studies examining host inflammation following the onset of obesity [21, 23]. In particular, the expression of IL-23, a key mediator of the inflammatory response in the gut, is upregulated in the ileum of the animals fed the HF diet. We also determined increased expression of genes encoding IL-1β in the ileum and liver, iNOS in the ileum and TNF-α in the liver [24, 25], all of which are associated with the onset of obesity. Whilst TNF-α is essential for anti-listerial resistance [24], the role of both IL-1β and iNOS during *Listeria* infection is less pronounced and subject to some debate [25–27]. Ileal expression of genes encoding anti-listerial cytokines IFN-γ [28] and IL-17 [29] was significantly reduced in HF mice prior to infection. Notably, reduced expression of *RegIII-γ* was more pronounced in HF diet-fed animals in comparison to LF- or chow-fed mice. RegIII-γ is an anti-bacterial lectin that is anti-listerial [30, 31] and also plays a role in microbial homeostasis in the gut through targeting Gram-positive commensals [32]. The expression of RegIII-γ and other anti-bacterial peptides are known to be influenced by diet and are subject to control by the microbiota [33].

Overall transcription of genes encoding tight junction proteins in the ileum was reduced in HF-fed animals relative to the other groups. Expression of tight junction proteins can be used as an assessment of barrier function and has previously been shown to be reduced in obese mice [34, 35]. Short-term studies in rodents demonstrate a reduction of claudin-7 levels following 4 weeks of HF diet [36], but no changes in Zo-1 at 3 days [37] or 1 week [35] of HF dietary feeding, suggesting time-dependent alterations which we see at 2 weeks of dietary intervention. Finally, expression of genes encoding mN-cad [8] and MET [38], known binding sites for *L. monocytogenes* invasion factors InlA and InlB in our murine model of infection, were not altered in the ileum of HF-fed mice but were significantly increased in the livers of these animals when compared to LF- or chow-fed groups. The gene encoding E-cad was downregulated in the ileum in concert with other epithelial junction proteins. Overall, we demonstrate significant alterations to the gastrointestinal environment through short-term HF feeding just prior to infection in our model system with many changes potentially relevant to the pathogenesis of *L. monocytogenes*.

### Short-term HF diet alters the gut microbiota

As the gastrointestinal microbiota is known to provide a barrier to *L. monocytogenes* infection [10] and also influences local barrier function [35] and immune homeostasis [1, 39, 40], we investigated alterations of the microbiota in our model at day 13 (prior to infection) with a focus upon differences between groups fed HF and matched LF diets. The extent of similarity between microbial communities was visualized through unweighted UniFrac PCoA of operational taxonomic units (OTUs), grouped at 97% sequence identity. The β-diversity metrics support a clear dietary driven separation (along PC2) between the HF-fed (in red) and LF-fed (in blue) mice on day 13 (Fig. 2c). α-diversity metrics are represented in Additional file 3: Figure S3. Relative to animals on a LF diet, the HF group had an increased representation of bacteria belonging to the *Firmicutes* phylum and a decrease in the *Bacteroidetes* (Fig. 2d; Additional file 4: Figure S4). This shift in the *Firmicutes/Bacteroidetes* ratio is associated with low-grade inflammation, reduction in barrier function and glucose intolerance in the context of a diet rich in animal fat [41]. Figure 2e shows a significant increase in the abundance of *Bacteroidaceae* and *Rikenellaceae* in the LF-fed group and a significant increase in abundance of *Ruminococcaceae* and *Lachnospiraceae* in the HF-fed mice. These patterns of family-level alterations to the microbiota have previously been reported for healthy individuals and patients with disorders associated with obesity, respectively [42, 43]. The data demonstrate significant diet-related alterations to gut microbiota composition upon short-term feeding prior to *Listeria* infection. Therefore, in our experimental model, *L. monocytogenes* is introduced into a gut environment in which there has been a considerable taxonomic shift influenced by diet and previously associated with reduction in barrier function.

To further investigate any potential links between microbiota changes and the host response to dietary feeding, we correlated changes in host gene expression with the abundance of individual members of the faecal microbiota at the genus level (Fig. 2f). *Butyricicoccus*, Clostridium XIVa, *Streptococcus* and *Mucispirillum* were more abundant in mice fed a HF diet, and their abundance correlated with induction of genes encoding host inflammatory responses. Associations between these genera and inflammatory conditions have been reported previously [40, 44]. *Parabacteroides* and *Bacteroides* were relatively more abundant in LF-fed mice and have previously been reported to be involved in the maintenance of immune homeostasis in the gut and maintenance of intestinal barrier integrity [45, 46]. Herein, we identified genera (e.g. Clostridium XI, Clostridium XIVa, *Enterococcus* spp.) which are influenced by HF dietary changes and are correlated with expression of genes encoding receptors for *L. monocytogenes* as well as genes associated with inflammation in the ileum and liver.

### High-fat diet alters the physiological response to *L. monocytogenes* infection

**Fig. 3:**
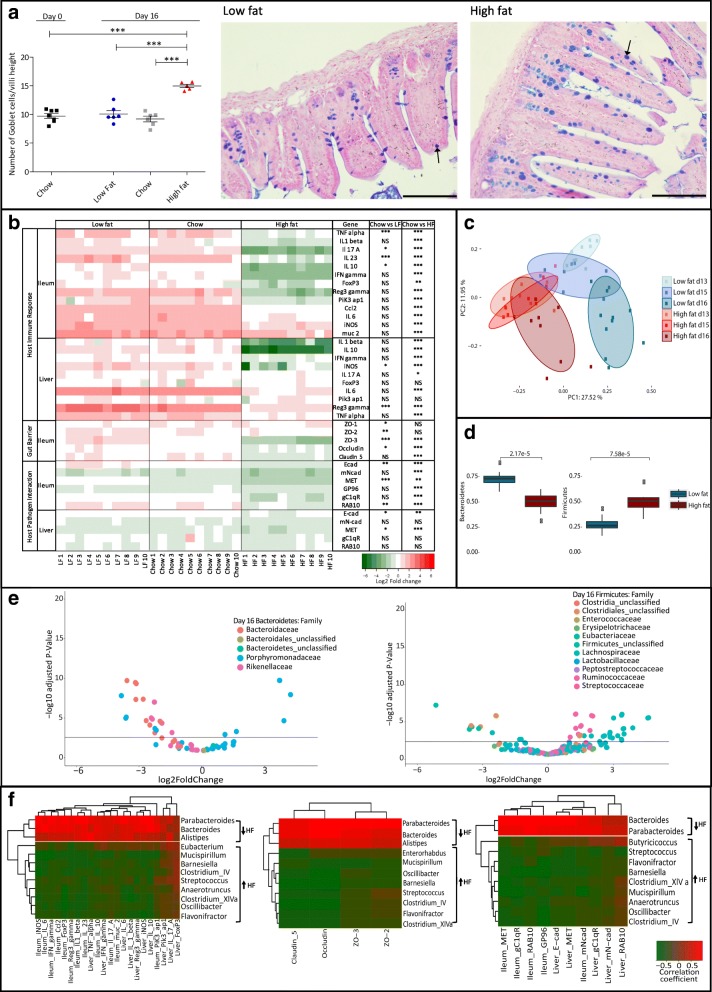
Impact of dietary fat content in the murine regulatory response to *L. monocytogenes* infection (D16 in relation to D13). **a** The number of goblet cells (D16) present on one villus was quantified and divided by the villus length (groups were blinded). Statistical analysis was conducted using one-way ANOVA and Dunnett’s multiple comparison test in relation to D0. Representative histological images demonstrating goblet cell density (left representative of LF group and right representative of HF group on day D16). Ileal paraffin sections of 5 μm were stained with alcian blue and periodic acid-Schiff (PAS) and counterstained with Schiff reagent and Nuclear Fast Red solution. Arrows indicate examples of goblet cells. **b** Effects of diet on host gene expression post-infection using qRT-PCR. Gene expression relative to D13 (pre-infection), within the same dietary group, in both ileum and liver on day 16 of dietary feeding (3 days post-infection). Represented as log_2_ of the fold change between the condition and the control (same diet D13). Statistical analysis was conducted using one-way ANOVA and Dunnett’s multiple comparison test. **c** Comparison of the changes in bacterial communities during controlled feeding. Unweighted Unifrac PCoA faecal microbiota distances between groups of mice fed different diets (blue representing LF; red representing HF) over indicated time points (D13, D15, D16). *p* values were measured using an Adonis test (*p* value = 0.00099, *R*^2^ is 0.148). **d** Changes in bacterial percentage of abundance during dietary shifts (D13 to D16) of the most abundant phyla, *Bacteroidetes* and *Firmicutes*. **e** Changes in bacterial abundance between LF and HF diets at the family level for the most abundant phyla. A volcano plot showing the fold change between high- and low-fat diets at D16. Each point represents an operational taxonomic unit (OTU). The *x*-axis represents in the log_2_ of the fold change whilst the *y*-axis is the negative log_10_ of DESeq2 *p* values adjusted for multiple testing using the false discovery rate method. Points to the right of the plot with positive log2FoldChange values represent bacterial taxa with increased abundance in the mice on the HF diet relative to the mice on the LF diet and those with negative log2FoldChange values represent bacterial taxa with increased abundance in the LF diet relative to the HF diet. The horizontal black line represents the cutoff for statistical significance, an adjusted *p* value of 0.05. **f** A correlation between the host regulatory response and the microbiota at the genus level on day 16. Spearman correlation, between the diet-dependent relative abundance of bacterial genera (arrows represent abundance in the mice on the HF diet relative to the mice on the LF diet) and the fold change for genes in both ileum and liver. Results shown separately for host immunity, tight junctions and host-pathogen interaction genes. Represented are only significant hits, *p* < 0.05. Error bars represent SEM

We subsequently determined the physiological response to oral *L. monocytogenes* EGDe^m^ infection at 3 days post-infection in the context of HF diet. Goblet cell numbers post-infection remained elevated in the ileum of HF diet-fed animals relative to LF- or chow-fed animals (Fig. 3a). Gene expression profiling of target genes was used to compare gene expression post-infection with the time point immediately prior to infection (day 13). Analysis revealed a reduction in expression of genes encoding inflammatory markers in both ileum and liver of HF diet-fed animals when compared to mice fed a LF diet or chow (Fig. 3b). This is supported by histological analysis of ileal tissue which indicated reduced immune cell infiltration in response to infection in mice fed a HF diet (Additional file 5: Figure S5). These are unexpected findings as these mice have a higher infectious load in local tissues relative to LF- or chow-fed animals. Furthermore, in our model, we observed that *Listeria* infection in the context of HF diet feeding also resulted in a further reduction in expression of genes encoding tight junction proteins suggestive of a further impairment of barrier function. Our findings are potentially reflective of very recent studies demonstrating that *L. monocytogenes* crosses the intestinal epithelial barrier by inducing significant mislocalization and reduction of expression of occludin, claudin-1 and E-cad, through the induction of TNF-α and IL-6 [17].

We appreciate that a shutdown of gene expression may, in some manner, be a consequence of higher numbers of the pathogen in the tissue, but to our knowledge, a dose-response correlating immune stimulation with increasing infectious load of *L. monocytogenes* has not been examined previously. We propose that the resultant dampening of immune stimulation in our model is a consequence of both the presence of the pathogen and increased dietary fat intake. In support of this, very recent work has shown that *Borrelia burgdorferi* infection in mice fed a high-fat diet suppresses innate immunity suggesting an immune-regulatory role of dietary fat intake which may favour infection [47].

The analysis of the microbiota during *L. monocytogenes* infection (Fig. 3c) indicates a clear separation of communities resulting from the presence of the pathogen in the context of diet. In particular, LF-fed animals undergo a profound rearrangement of the microbial community structure from D13 to D16 as a consequence of infection, in both principal components. An increased *Firmicutes/Bacteroidetes* ratio in the HF group is maintained during *L. monocytogenes* infection (Fig. 3d). The microbial families affected by infection (D16) (Fig. 3e; Additional file 4: Figure S4) resemble those that are influenced by diet alone (D13) (Fig. 2e); however, we see a stronger representation of the numbers of operational taxonomic units (OTUs) for both *Bacteroidaceae* and *Rikenellaceae* in the LF-fed group and *Ruminococcaceae* and *Lachnospiraceae* in the HF-fed mice following *Listeria* infection. This suggests that *Listeria* infection in the context of HF diet potentially amplifies OTUs associated with diet-induced inflammation. Interestingly, the representation of the *Clostridiales* family as indicated by the number of significant OTUs has increased abundance in the LF-fed group. This family has recently been associated with *L. monocytogenes* clearance upon infection [10].

Correlating microbial genera with host gene expression (Fig. 3f) highlights the general downregulation of host gene expression following *Listeria* infection in the HF-fed group. Directional changes to specific microbial genera influenced by HF diet (Fig. 3f) were similar to those seen to be induced by diet alone (Fig. 2f). However, the correlations with associated host gene expression profiles were generally reversed, indicating that *L. monocytogenes* infection in the context of HF diet was a significant negative modulator of selected host genes.

### High-fat diet increases susceptibility to systemic *L. monocytogenes* infection

**Fig. 4:**
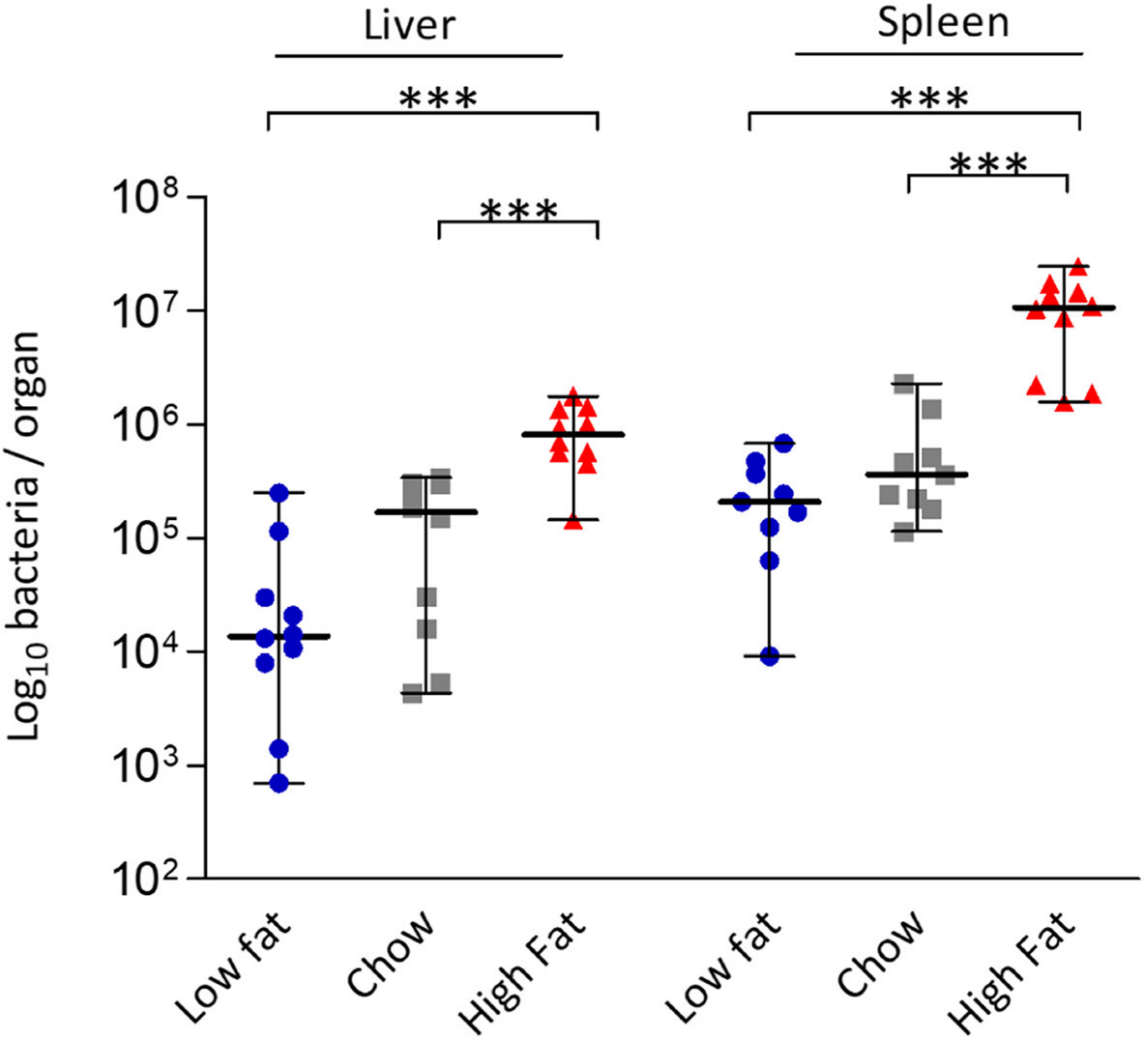
Increased dietary fat increases host susceptibility to systemic infection with *L. monocytogenes* EGDe^m^. Bacterial burden of *Listeria* in the spleen and liver of C57BL/6 mice fed for 13 days with different diets and subsequently infected via the IP route (*n* = 10). Standard deviation from the mean, statistical analysis was conducted using one-way ANOVA and Dunnett’s multiple comparison test in relation to chow diet. ***p < 0.001. Error bars represent SEM

Whilst this study revealed that diet alone is a driver of physiological changes in the ileum, it also highlighted an unexpected increase in expression of genes encoding *L. monocytogenes* binding sites (including *E-Cad*, *N-Cad*, *gC1qR*) in the liver (Fig. 2b). We also noted alterations to expression levels of genes encoding cytokines in the liver that have the potential to influence resistance to infection, including an increase in transcription of the anti-inflammatory cytokine IL-10 in animals fed a HF diet (Fig. 2b). As these changes occurred prior to infection, we went on to determine the influence of diet upon the systemic phase of infection. An intraperitoneal (IP) infection of *L. monocytogenes* EGDe^m^ was administered after 2 weeks of dietary modulation (Fig. 4). The results reveal a clear influence of HF diet upon the systemic phase of *L. monocytogenes* compared to both chow and LF diets. The phenomenon was seen for both the murinized EGDe^m^ and wild-type EGDe strains (Additional file 6: Figure S6) of *L. monocytogenes*, suggestive that this effect is not solely due to an increase in mN-Cad. The data suggest that a westernized HF diet alters the physiology of the host beyond the gut to heighten susceptibility to infectious disease.

## Conclusions

In the context of a global obesity epidemic and changes in dietary habits towards increased consumption of a ‘westernized’ diet, there is currently surprisingly little information regarding the influence of diet upon the progression of infectious disease. Herein, we demonstrate that a HF westernized diet significantly and reproducibly increases susceptibility to *L. monocytogenes* in a murine model. HF dietary feeding prior to the onset of obesity influenced parameters in mice that impact both the intestinal and systemic phase of infection, suggesting a profound system-wide alteration in host physiology. We appreciate that we have not examined alterations to listerial expression of virulence factors that may occur as a result of luminal alterations of nutrients. For instance, altered responses in *Listeria* have been reported in response to exposure to various fatty acids in vitro [48] and may therefore have the potential to influence infectivity. It is interesting to note that other studies have demonstrated that a high-fat food delivery matrix can increase infectivity of *L. monocytogenes* in murine [49] or primate [50] models. However, the physiological effects on the host of transient high-fat feeding were not considered in those studies. The data presented herein support emerging evidence that diet can significantly influence infectious disease models [51, 52] and suggest that diet should be a factor in future evaluation of the infectious dose of the pathogen. The work raises the intriguing possibility that a westernized diet may be a significant factor influencing host resistance to infection.

## Methods

### Animal dietary intervention and infection

Seven-week-old female C57BL/6 mice (ENVIGO, UK), *n* = 10, were housed in a controlled environment with free access to food and water. The mice were fed a control chow diet (Teklad Global 2018S Rodent Diet, ENVIGO, UK), a low-fat diet (DIO series diets D12450H, Research Diets, Inc., USA) or a high-fat (DIO series diets D12451, Research Diets, Inc., USA) for 13 days. Thereafter, the animals were either infected through oral inoculation (IG) or intraperitoneal (IP) infection . Overnight culture of the murinized strain, *L. monocytogenes* EGD-e InlA^*m*^, was centrifuged (7000×*g* for 5 min), washed twice with PBS and resuspended in PBS. A 200-μl inoculum comprised 3.2 × 10^9^ CFU for the IG infection and 9 × 10^4^ for IP infection. The progression of infection followed over a 3-day period [13, 53], with mice maintained on their specific diets for that period. Mice were euthanized, and the internal organs aseptically removed and homogenized using stomacher bags and PBS. For CFU per organ enumeration, dilutions were plated on BHI (brain heart infusion) agar plates. Following IG infection, on days 14 (day 1 post-infection) and 16 (day 3 post-infection), the faecal pellets were collected and plated for CFU to determine shedding of *L. monocytogenes*.

### RNA extraction and quantitative RT-PCR analysis

A sample of the liver and the ileum were collected for the analysis of the host regulatory response to both diet and infection (transcriptome analysis). The samples were stabilized with RNAlater (Sigma) and stored at − 80 °C until total RNA extraction (RNeasy Plus Universal Mini Kit, Qiagen). The total RNA extracted was DNase treated (TURBO DNA-free™ Kit, Ambion), and the transcriptomic analysis was done using RT-PCR (Transcriptor Reverse Transcriptase, Roche). qPCR protocol was carried out using LightCycler® 480 Probes Master (Roche) with the Universal Probe Library from Roche. Primers are outlined in Additional file 7: Table S1. The amplification setup used was 45 runs in 384-well plates with the MonoColor hydrolysis probe detection format.

### Faecal samples and microbiota profiling

For the analysis of mouse gut microbiota based on 16S rRNA gene amplicon sequencing, faecal samples were collected during dietary adaptation prior to infection (on days 0, 6 and 13) and after infection (on days 15 and 16). Pellets collected were used for microbiota analysis using 16S rDNA sequencing (DNA extraction using QIAmp fast DNA stool mini Kit, Qiagen). The quality of raw sequences was visualized with FastQC. This was followed by quality filtering using trimmomatic [54]. Briefly, the first twenty and last twenty bases were trimmed and then a sliding window was applied (window size 4 with minimum quality 15) along with a minimum sequencing length of 250 bases. Subsequently, sequences were filtered via USEARCH with a maximum e score of 1 [55], and following this, open reference operational taxonomic unit (OTU) clustering was performed in qiime against the Ribosomal Database Project (RDP) database v11.4 [56]. For sequences failing to be clustered against the reference database, de novo clustering was applied [57]. Chimeric sequences were removed with ChimeraSlayer with the Gold database and UCHIME [58]. The resulting OTU sequences were classified, phylum to genus, using Mothur [59] and the RDP database v11.4 with any assignments with a bootstrap value of less than 80% labelled unclassified. Species classification was carried out using SPINGO (v1.3) against the RDP database (v11.4) with default parameters (similarity score of 0.05 and a bootstrap cutoff of 0.8) [60]. All downstream analysis was performed in R version 3.4.3. Alpha and beta diversity was calculated using the R package phyloseq [61]. Differences in alpha diversity were assessed using the Mann-Whitney test. Differential abundant analysis was performed using DESeq2 [62]. The adonis function in the vegan library was used to assess group-level differences in the microbiota.

### Tissue staining and microscopy

Ileal samples were collected for histology analysis, and the identity of sample groups was blinded to the investigator. The tissue sample was stored in 4% paraformaldehyde for 24 h at room temperature and dehydrated with 70% ethanol for 72 h at 4 °C, prior to paraffin embedding (dehydration and permeation in molten wax in the histokinette in a 21 h overnight cycle; wax blocking of the samples using the console system TissueTek for 2 h). The wax moulds containing the embedded tissue were cut using the Leica RM2135 rotary Microtome. For goblet cell analysis, ileal paraffin sections of 5 μm were stained with alcian blue and periodic acid-Schiff (PAS) and counterstained with Schiff reagent and Nuclear Fast Red solution. Sections were mounted in DPX mounting reagent (Sigma) and imaged using the Olympus BX51 microscope (Olympus DP71 camera), with a × 20 objective. Image analysis was performed using ImageJ. For histological scoring, paraffin-embedded ileal sections (5 μm) were stained with haematoxylin and eosin according to standard procedures. The sections were blindly scored using a light microscope (Olympus BX51, Olympus, Germany). The histology score was adapted from Drolia et al. [17] with some modifications. The ileal samples were scored on a scale of 0–3 for two parameters: infiltration of inflammatory cells (mostly mononuclear cells) to the villi and infiltration of mono- and polymorphonuclear cells to the crypt, yielding a maximum score of 6. In our model, polymorphonuclear cells were mainly located at the bottom of the crypts. The gradient of the inflammatory cell infiltration was based on 3 = highly increased, 2 = moderately increased, 1 = mildly increased and 0 = normal.

### Statistical analysis

Statistical analyses were conducted with Prism 5 (Graph-Pad Software). Mann-Whitney test was used to compare the means of the two groups. One-way ANOVA with a Dunnett’s multiple comparison test was used for pair-wise comparison of means from more than two groups in relation to the control, or with Tukey’s post hoc test for comparison of means relative to the mean of a control group.

If the *p* value falls above 0.05, the mean differences were considered statistically non-significant (NS). For statistically significant differences, *p < 0.05, **p < 0.01 and ***p < 0.001.

## Additional files

Additional file 1: **Figure S1.** Diet composition and murine weights prior to oral infection. (PDF 306 kb)

Additional file 2: **Figure S2.** Increased dietary fat from animal source increases host susceptibility to oral infection with *Listeria monocytogenes* EDGe^m^ pIKM2 (PDF 413 kb)

Additional file 3: **Figure S3.** Assessment of diversity within the sample (Alpha diversity) in the groups fed high-fat or low-fat diets. (PDF 498 kb)

Additional file 4: **Figure S4.** β-diversity metrics. (PDF 434 kb)

Additional file 5: **Figure S5.** Histopathology score. (PDF 490 kb)

Additional file 6: **Figure S6.** Increased dietary fat compromises the host systemic immune response and increases host susceptibility to IP infection with wild-type *L. monocytogenes* EGDe. (PDF 355 kb)

Additional file 7: **Table S1.** Transcriptome analysis primer sets. (PDF 370 kb)
