# Supplementary material for: Short-term consumption of a high-fat diet increases host susceptibility to Listeria monocytogenes infection
Source: Microbiome. 2019 Jan 18;7:7. doi: 10.1186/s40168-019-0621-x (PMC6339339; doi:10.1186/s40168-019-0621-x)
Supplement: Supplementary file 1 — Figure S1. Diet composition and murine weights prior to oral infection. (PDF 306 kb) [file 40168_2019_621_MOESM1_ESM.pdf]

Supplemental data, Las Heras et al *Fig S1.*

A

	Low fat diet		High fat diet	
	**gm%**	**kcal%**	**gm%**	**kcal%**
**Protein**	19.2	20	24	20
**Carbohydrate**	67.3	70	41	35
**Fat**	4.3	10	24	45
**Total**	**100**		**100**	
**kcal/gm**	**3.85**		**4.73**	
**Ingredient**	**gm**	**kcal**	**gm**	**kcal**
Casein, 30 Mesh	200	800	200	800
L-Cystine	3	12	3	12
Corn Starch	452.2	1808.8	72.8	291
Maltodextrin 10	75	300	100	400
Sucrose	172.8	691.2	172.8	691
Cellulose, BW200	50	0	50	0
Soybean Oil	25	225	25	225
Lard	20	180	177.5	1598
Mineral Mix S10026	10	0	10	0
DiCalcium Phosphate	13	0	13	0
Calcium Carbonate	5.5	0	5.5	0
Potassium Citrate, 1 H2O	16.5	0	16.5	0
Vitamin Mix V10001	10	40	10	40
Choline Bitartrate	2	0	2	0
**Total**	1055.05	4057	858.15	4057

B

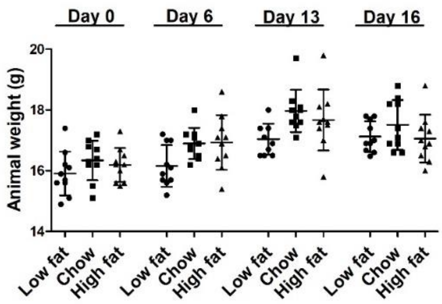

Figure S1. Diet composition and murine weights prior to oral infection. A. Composition of experimental diets. **B.** Variation of animal weight over time with dietary change. Statistical analysis was conducted using One way ANOVA and Tukey's Multiple Comparison Test.
