# Supplementary material for: Short-term consumption of a high-fat diet increases host susceptibility to Listeria monocytogenes infection
Source: Microbiome. 2019 Jan 18;7:7. doi: 10.1186/s40168-019-0621-x (PMC6339339; doi:10.1186/s40168-019-0621-x)
Supplement: Supplementary file 2 — Figure S2. Increased dietary fat from animal source increases host susceptibility to oral infection with Listeria monocytogenes EDGem pIKM2 (PDF 413 kb) [file 40168_2019_621_MOESM2_ESM.pdf]

Supplemental data, Las Heras et al *Fig S2.*

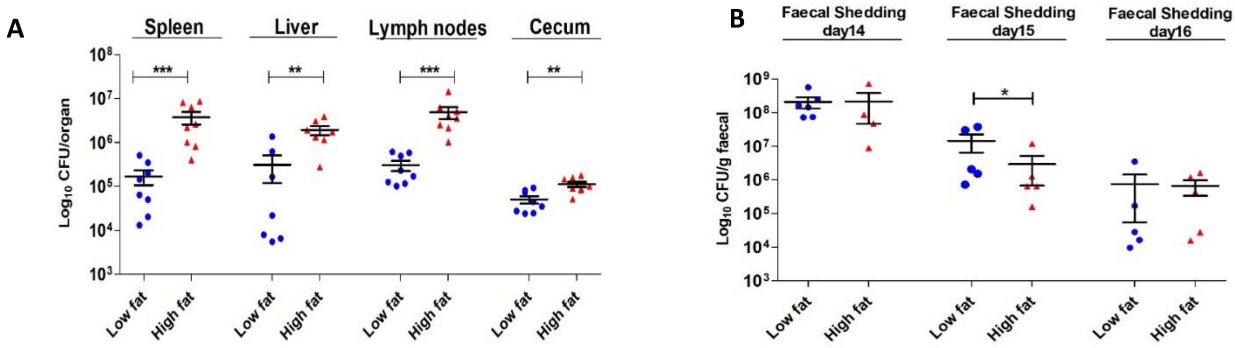

C.

Organs	Low fat	High fat
Spleen	p=0.7618 (NS)	p=0.0205 (*)
Lymph nodes	p=0.5049 (NS)	p=0.9654 (NS)
Cecum	p=0.1457 (NS)	p=0.2766 (NS)
Liver	p=0.3148 (NS)	p=0.0001 (***)

Figure S2. Increased dietary fat from animal source increases host susceptibility to oral infection with *Listeria monocytogenes* EDGe^{m} pIKM2. IG inoculation of seven-week-old female C57BL/6 mice (ENVIGO, UK) was repeated in a separate duplicate experiment (n=8) using a 200µl inoculum comprising 2.8×10^{9} CFU *L. monocytogenes* EGDm*::pIMK2lux*, a bioluminescent murinized strain. After infection (three days), the faecal pellets were collected daily and plated for CFU to determine shedding of *L. monocytogenes.* **A.** Bacterial burden of spleen, liver, lymph nodes and of C57BL/6 mice fed with diets varying in percentage of fat content from the total caloric intake (n=8, standard deviation from the mean, statistical analysis was conducted using Mann Whitney Nonparametric Test). **B.** *Listeria monocytogenes* bacterial shedding per gram of faecal sample in each day after infection. **C.** Comparison between the two trials (EGDe^{m} and pIKM2). Mann Whitney Nonparametric Test.
