# Supplementary material for: Short-term consumption of a high-fat diet increases host susceptibility to Listeria monocytogenes infection
Source: Microbiome. 2019 Jan 18;7:7. doi: 10.1186/s40168-019-0621-x (PMC6339339; doi:10.1186/s40168-019-0621-x)
Supplement: Supplementary file 3 — Figure S3. Assessment of diversity within the sample (Alpha diversity) in the groups fed high-fat or low-fat diets. (PDF 498 kb) [file 40168_2019_621_MOESM3_ESM.pdf]

Supplemental data, Las Heras et al *Fig S3.*

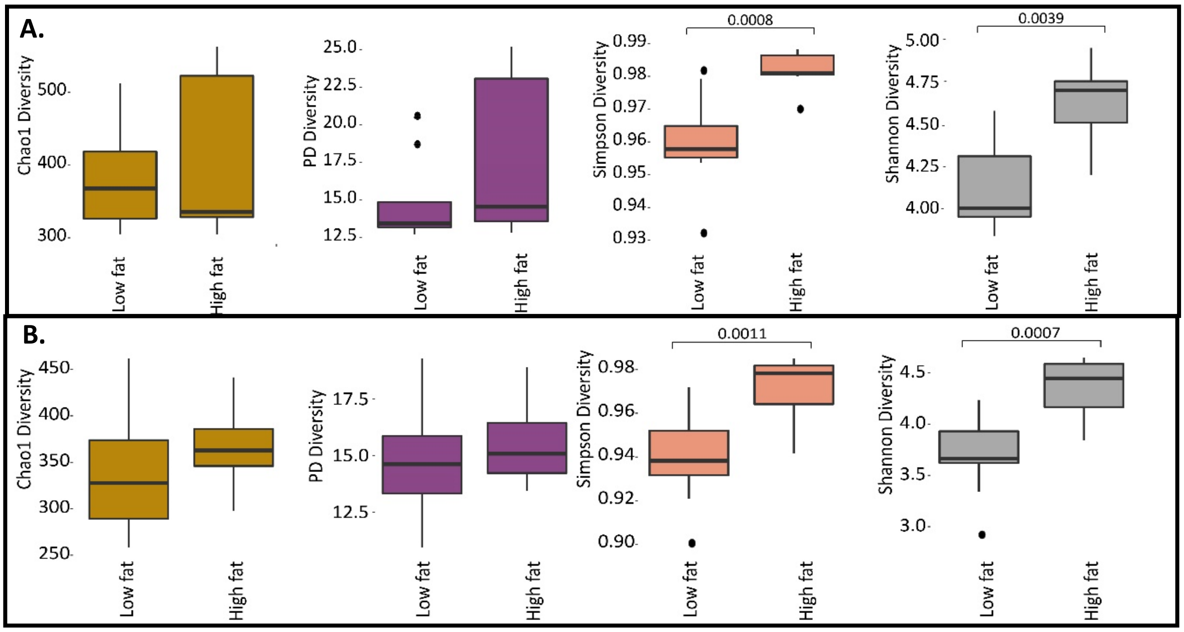

Figure S3. Assessment of diversity within the sample (Alpha diversity) in the groups fed high-fat or low-fat diets. Chao1 as a measurement of sample coverage. PD (phylogenetic diversity) used to consider affiliation between taxa within diversity. Simpson and Shannon used as an estimation of richness and evenness respectively. **A.** Changes in intestinal microbiota communities caused by short-term increased dietary fat (D0 to D13). **B.** Changes in intestinal microbiota communities upon infection with *L. monocytogenes*, modulated by short-term increased dietary fat (D13 to D16).
