# Supplementary material for: Short-term consumption of a high-fat diet increases host susceptibility to Listeria monocytogenes infection
Source: Microbiome. 2019 Jan 18;7:7. doi: 10.1186/s40168-019-0621-x (PMC6339339; doi:10.1186/s40168-019-0621-x)
Supplement: Supplementary file 4 — Figure S4. β-diversity metrics. (PDF 434 kb) [file 40168_2019_621_MOESM4_ESM.pdf]

Supplemental data, Las Heras et al *Fig S4.*

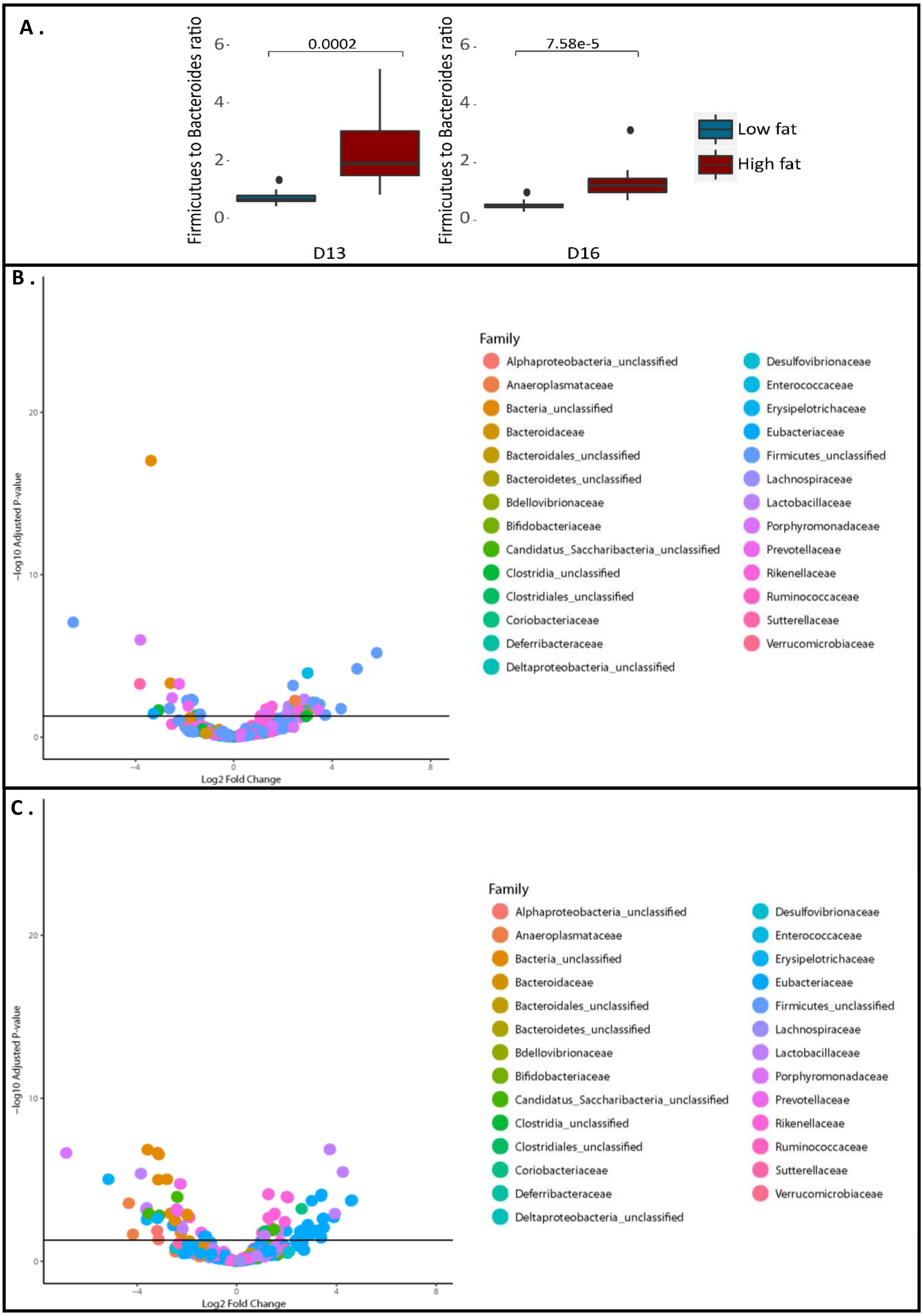

Figure S4. β-diversity metrics. A. Changes in Firmicutes to Bacteroidetes bacterial ratio of abundancy during dietary shifts (D13 and D16). **B and C.** Changes in total bacterial abundancy between low and high-fat diets at the family level. A volcano plot showing the fold change between high and low fat diets at (B. D13 and C. D16). Each point represents an Operational Taxonomic Unit (OTU). The X axis represents in the log2 of the fold change while the Y axis is the negative log10 of DESeq2 P values adjusted for multiple testing using the False Discovery Rate method. Points to the right of the plot with positive log2FoldChange values represent bacterial taxa with increased abundancy in the mice on the high fat diet relative to the mice on the low fat and those with negative log2FoldChange values represent bacterial taxa with increased abundancy in the low fat diet relative to the high fat diet. The horizontal black line represents the cut off for statistical significance, an adjusted p-value of 0.05.
