# Supplementary material for: Short-term consumption of a high-fat diet increases host susceptibility to Listeria monocytogenes infection
Source: Microbiome. 2019 Jan 18;7:7. doi: 10.1186/s40168-019-0621-x (PMC6339339; doi:10.1186/s40168-019-0621-x)
Supplement: Supplementary file 5 — Figure S5. Histopathology score. (PDF 490 kb) [file 40168_2019_621_MOESM5_ESM.pdf]

Supplemental data, Las Heras et al. *Fig. S5*

A

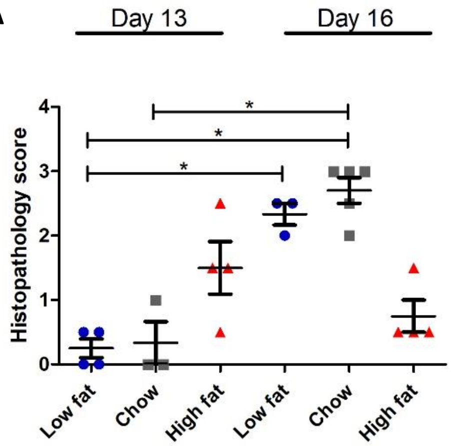

B

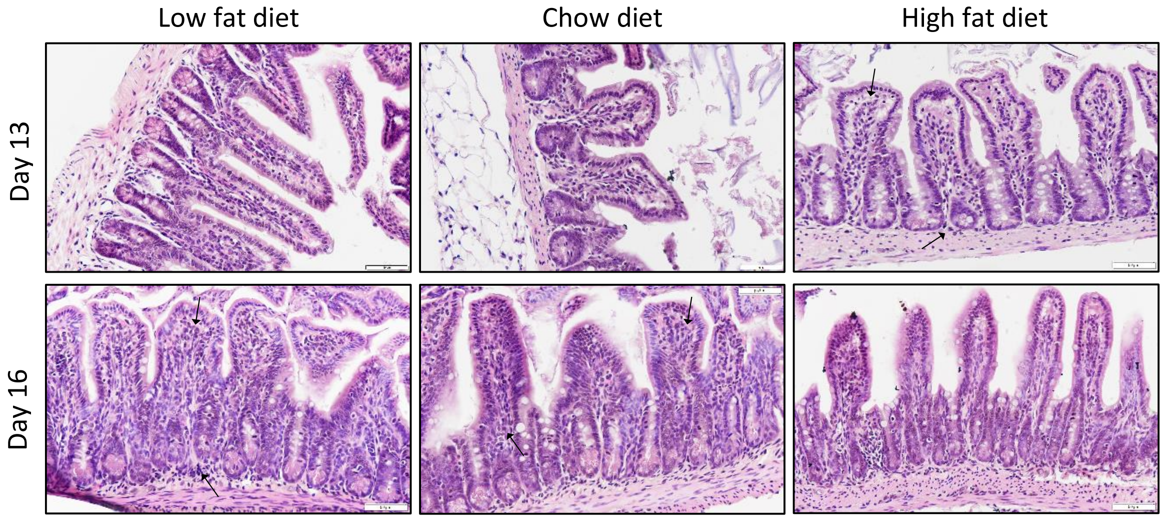

Figure S5. Histopathology score. A. Histopathology score of ileal sections 13 days post dietary change (Day 13) and 3 days post-infection (Day 16). Error bars represent SEM. **B.** The ileal samples were scored on a scale of 0-3 for 2 parameters: infiltration of inflammatory cells (mostly mononuclear cells) to the villi and infiltration of mono- and polymorphonuclear cells to the crypt, yielding a maximum score of 6. In our model, polymorphonuclear cells were mainly located at the bottom of the crypts. The gradient of the inflammatory cell infiltration was based on: 3 = highly increased; 2 = moderately increased, 1 = mildly increased and 0 = normal (scale bar, 50μm).
