# Supplementary material for: Short-term consumption of a high-fat diet increases host susceptibility to Listeria monocytogenes infection
Source: Microbiome. 2019 Jan 18;7:7. doi: 10.1186/s40168-019-0621-x (PMC6339339; doi:10.1186/s40168-019-0621-x)
Supplement: Supplementary file 6 — Figure S6. Increased dietary fat compromises the host systemic immune response and increases host susceptibility to IP infection with wild-type L. monocytogenes EGDe. (PDF 355 kb) [file 40168_2019_621_MOESM6_ESM.pdf]

Supplemental data, Las Heras et al., *Fig. S6*

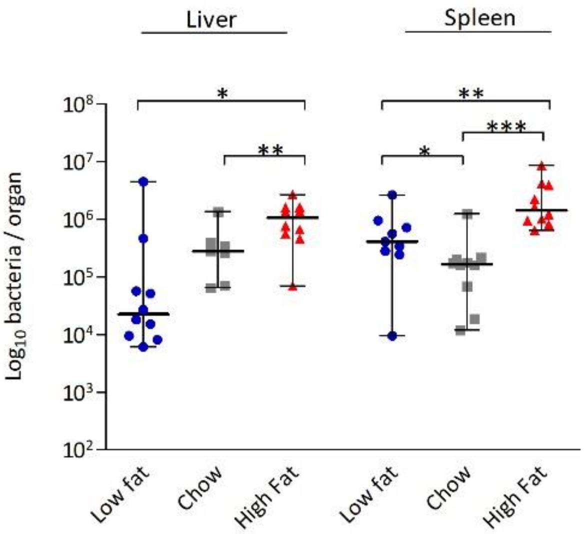

Supplementary Fig S6. Increased dietary fat compromises the host systemic immune response and increases host susceptibility to IP infection with wild-type *L. monocytogenes* EGDe. Bacterial burden in spleen and liver (n=10, standard deviation from the mean, statistical analysis was conducted using One way ANOVA and Dunnett Multiple Comparison Test Test in relation to chow diet). C57BL/6 mice fed for 13 days with diets varying in percentage of fat content from the total caloric intake and thereafter infected ip with $5x10^4$ (LF and Chow groups) or $1x10^4$ (HF group) *L. monocytogenes EGDe*. Animals were euthanized at 72 nours post-infection and the total number of *L. monocytogenes EGDe* CFUs per organ were determined by plating homogenized organs.
