# Supplementary material for: Short-term consumption of a high-fat diet increases host susceptibility to Listeria monocytogenes infection
Source: Microbiome. 2019 Jan 18;7:7. doi: 10.1186/s40168-019-0621-x (PMC6339339; doi:10.1186/s40168-019-0621-x)
Supplement: Supplementary file 7 — Table S1. Transcriptome analysis primer sets. (PDF 370 kb) [file 40168_2019_621_MOESM7_ESM.pdf]

Supplemental data, Las Heras et al *Table S1.*

Supplementary Table S1. Transcriptome analysis primer sets:

**Gene**			**Forward primer (left)**	**Reverse primer (right)**	**Probe**
**House Keeping (Ileum)**	B-actin	*Actb*	aaggccaaccgtgaaaagat	gtggtacgaccagaggcatac	#56
	Rib-S18	*Rps18*	aagcagacatcgacctcacc	ggaaccagtctgggatcttg	#88
**House Keeping (Liver)**	Trypsin inhibitor	*Itih4*	cccctggacctcctcttg	agcaggatattgtgctgcaa	#21
	microglobulin/bikunin	*ambp*	ccactaataactgggaccctca	ggggccttctgagtaattga	#51
**Host cell invasion**	Ecad	*Cdh1*	atcctcgccctgctgatt	accaccgttctcctccgta	#18
	Met	*Ryk*	ggcatgcaaagtccaagg	catgcccatagccacaaagt	#33
	GP96	*Grp94*	aatgaggagttaacggtcaagatt	ttcctacacccgtgtctgtg	#62
	gC1qR	*C1qbp*	aggaggagccctcacagg	cacaaagttgggagttgatgtc	#92
	RAB10	*Rab10*	gaaaaggcgttcctcacatt	tgggttcttttacaggggtct	#88
	mNcad	*Cdh2*	gccatcatcgctatccttct	ccgtttcatccataccacaaa	#18
**Permeability**	ZO-1	*Tjp1*	gagcctgctaagccagtcc	cagcatcagtttcgggtttt	#21
	ZO-2	*Tjp2*	tcatcaaagaaatgaccagaaca	tctcagtaacagtgccgttga	#49
	ZO-3	*Tjp3*	aggccgtgcagtttctgt	gcgagactggaccatcttct	#16
	Occludin	*Ocln*	ggtctctacgtggatcaatatttgta	aaccccaggacaatggcta	#79
	claudins	*Ocld1*	cccttgacccccatcaat	acacctcccagaaggcaga	#20
**Immune response**	TNF-α	*TNFg*	tcttctcattcctgcttgtgg	ggtctgggccatagaactga	#49
	IL1-β	*IL1b*	ttgacggaccccaaaagat	agctggatgctctcatcagg	#38
	IL17A	*IL17a*	gattttcagcaaggaatgtgg	cattgtggagggcagacaat	#34
	IL23	*IL23*	tccctactaggactcagccaac	tgggcatctgttgggtct	#19
	INF-ɣ	*Infg*	atctggaggaactggcaaaa	ttcaagacttcaaagagtctgaggta	#21
	FoxP3	*Foxp3*	tcaggagcccaccagtaca	tctgaaggcagagtcaggaga	#78
	IL10	*IL10*	cagagccacatgctcctaga	gtccagctggtcctttgttt	#41
	IL6	*IL6*	gctaccaaactggatataatcagga	ccaggtagctatggtactccagaa	#6
	Reg3-ɣ	*Reg3g*	accatcaccatcatgtcctg	ggcatctttcttggcaactt	#108
	pIK3-ap1	*Pik3r1*	gacggcactttccttgtcc	tgacttcgccgtctaccac	#80
	Ccl2	*Ccl2*	catccacgtgttggctca	gatcatcttgctggtgaatgag	#62
	iNOS	*Impdh2*	tcttacgtgccggacgac	cccaggaagaatgagaaaatcat	#100
	Muc2	*Muc2*	gcagccacagatcccaaa	gttggggtcccctgaagt	#33

Primers for host transcriptome analysis. Designed in the Assay Design Center from Roche *Universal ProbeLibrary* (version 2.52, 2016).
